# Spermidine Synthase and Saccharopine Reductase Have Co-Expression Patterns Both in Basidiomycetes with Fusion Form and Ascomycetes with Separate Form

**DOI:** 10.3390/jof9030352

**Published:** 2023-03-14

**Authors:** Yayong Yang, Lei Shi, Xinyu Xu, Jin Wen, Tianyue Xie, Hui Li, Xiaoyu Li, Mengyu Chen, Xinyi Dou, Chengjin Yuan, Hanbing Song, Baogui Xie, Yongxin Tao

**Affiliations:** 1 College of Horticulture, Fujian Agriculture and Forestry University, Fuzhou 350002, China; 2 Mycological Research Center, Fujian Agriculture and Forestry University, Fuzhou 350002, China; 3 Institute of Cash Crops, Hebei Academy of Agriculture and Forestry Sciences, Shijiazhuang 050051, China

**Keywords:** chimeric gene, lysine, spermidine, basidiomycetes, ascomycetes

## Abstract

Gene fusion is a process through which two or more distinct genes are fused into a single chimeric gene. Unlike most harmful fusion genes in cancer cells, in this study, we first found that spermidine synthetase- (SPDS, catalyst of spermidine biosynthesis) and saccharopine reductase- (SR, catalyst of the penultimate step of lysine biosynthesis) encoding genes form a natural chimeric gene, *FfSpdsSr*, in *Flammulina filiformis*. Through the cloning of full-length ORFs in different strains and the analysis of alternative splicing in developmental stages, *FfSpdsSr* has only one copy and unique transcript encoding chimeric SPDS-SR in *F. filiformis*. By an orthologous gene search of *SpdsSr* in more than 80 fungi, we found that the chimeric *SpdsSr* exists in basidiomycetes, while the two separate *Spds* and *Sr* independently exist in ascomycetes, chytridiomycetes, and oomycetes. Further, the transcript level of *FfSpdsSr* was investigated in different developmental stages and under some common environmental factors and stresses by RT-qPCR. The results showed that *FfSpdsSr* mainly up-regulated in the elongation stage and pileus development of *F. filiformis*, as well as under blue light, high temperature, H_2_O_2_, and MeJA treatments. Moreover, a total of 15 sets of RNA-Seq data, including 218 samples of *Neurospora crassa*, were downloaded from the GEO database and used to analyze the expression correlation of *NcSpds* and *NcSr*. The results showed that the separate *NcSpds* and *NcSr* shared highly similar co-expression patterns in the samples with different strains and different nutritional and environmental condition treatments. The chimeric *SpdsSr* in basidiomycetes and the co-expression pattern of the *Spds* and *Sr* in *N. crassa* indicate the special link of spermidine and lysine in fungi, which may play an important role in the growth and development of fruiting body and in response to the multiple environmental factors and abiotic stresses.

## 1. Introduction

Gene fusion is a process by which the complete or partial sequences of two or more distinct genes are fused into a single chimeric gene or transcript as a result of DNA- or RNA-derived rearrangements [1]. Gene fusion could increase the diversity of gene functions and drive the evolution of organisms. However, natural gene fusion often leads to genomic disorders or cancer, leaving devastating consequences [2], because it can change the properties of precursor proteins and even perturb normal regulatory pathways so that the chimeric genes are often used as molecular diagnostic markers to detect and treat cancer in medicine [3,4].

As for the normal natural chimeric genes (rarely found), only the following cases have been reported in fungi. In *Metakinetoplastina protists*, the native fusion gene *SCAMK* formed through the insertion of a calmodulin-like III gene into the sucrose non-fermenting related kinase3 gene by an unequal crossover of homologous chromosomes in meiosis [5]. In *Penicillium brevicompactum*, Cytochrome P450 (MpaD) and a Hydrolase (MpaE) naturally fused to become the chimeric enzyme gene *MpaDE*, which is involved in the synthesis of mycophenolic acid (MPA) [6]. The *SPE3* gene (encoding spermidine synthase) and *LYS9* gene (encoding saccharopine dehydrogenase) were first found to be fused *SPE3-LYS9* in *Cryptococcus neoformans*, which encodes functional spermidine synthase and saccharopine dehydrogenase gene products [7]. However, in *Saccharomyces cerevisiae*, the spermidine synthase gene and saccharopine dehydrogenase gene were identified as independent *Spe3* and *LYS9*, respectively [8]. This indicates that the fusion of these two genes depends on the species. Whether the fusion behavior of these two different genes (*Spe* and *Lys*) is evolutionarily formed will need to be systematically analyzed in multiple species.

The function and effect of the formation of the normal natural chimeric genes, including SPE-LYS, has a lack of in-depth research. In *C. neoformans*, the spe3-LYS9 mutants (the SPE3-homologous region was replaced by the *NAT1* gene cassette) showed reduced capsule and melanin production and growth rate, and the SPE3-lys9 mutants (the LYS9-homologous region was replaced by the *NAT1* gene cassette) grew slowly and died upon lysine starvation, while the spe3-lys9 mutants (both the SPE3 and LYS9-homologous region were replaced by the *NAT1* gene cassette) were avirulent and unable to survive in vivo [7]. Due to the biosynthesis of lysine by higher fungi (basidomycetes and ascomycetes), it adopts the α-aminoadipate (AAA) pathway, in which the saccharopine dehydrogenase (also known as saccharopine reductase) catalyzes the penultimate step of the lysine biosynthesis [9,10,11]. This suggests that the chimeric SPE3-LYS9 gene may link spermidine and lysine biosynthesis, and their close connection is important for the growth and metabolism of *C. neoformans*.

Spermidine, as one of the main polyamines, is ubiquitous to all living organisms and involved in many fundamental cellular processes [12]. It has been reported that spermidine plays a role in regulating the growth, development, and secondary metabolism of fungi and participates in response to various abiotic stresses, such as high temperature, cold injury, drought, osmotic stress, oxidative stress and photooxidative damage, as well as jasmonic acid and abscisic acid induction [13]. Lysine, as a basic positively charged amino acid, plays an important physiological role in the organism and also functions in the growth, development, and response to stress in fungi [14]. The deficiency of lysine in fungi will not only damage protein synthesis but also seriously affect the immune system. In *Magnaporthe oryzae*, the deletion of the lysine synthase gene affects its growth, conidia germination, and pathogenicity in rice blast [15]. In *Flammulina filiformis*, higher levels of lysine content can facilitate stipe elongation and increase yield [16]. Thus, both spermidine and lysine are necessary for cell growth, proliferation, and differentiation, and they play an important role in maintaining normal physiological function and metabolism. Due to their important roles, there is a possibility that it is necessary to make spermidine and lysine act synchronously in many cases during the process of evolution, as some species fused their key biosynthesis catalytic enzyme together under natural conditions. Therefore, it is necessary to investigate the natural fusion phenomenon of spermidine synthase and saccharopine dehydrogenase in multiple species and summarize the conditions and circumstances when they are highly expressed, so as to better reveal the biological significance of their fusion.

In this study, we investigated more than 80 species from four common fungal groups (basidiomycetes, ascomycetes, chytridiomycetes and oomycetes) to explore the different forms of being (chimeric pattern or separate pattern) of spermidine synthase (SPDS) and saccharopine reductase (SR, the penultimate step of the lysine biosynthesis in fungi, equivalent to saccharopine dehydrogenase). Further, we selected *F. filiformis* (large agaric fungi and industrially produced edible fungi) as the representative species of basidiomycetes for research in this study because it has the typical morphological and developmental characteristics of fruiting body, it publishes the complete genome and transcriptome data of different developmental stages, and it has been widely studied in recent years. *Neurospora crassa*, as the representative species of ascomycetes, was also used for research in this study, because it has detailed genome maps and abundant expression profile data uploaded in the NCBI database. The structure, existence forms, and locus in the chromosome of *Spds* and *Sr* in *F. filiformis* and *N. crassa* were investigated and studied in detail. Moreover, the expression patterns of *SpdsSr* in *F. filiformis* and the separate *Spds* and *Sr* in *N. crassa* were analyzed to reveal the possible functional effects and mechanisms of the natural chimeric *SpdsSr* pattern in basidiomycetes and their separate but cooperative expression pattern in ascomycetes.

## 2. Materials and Methods

### 2.1. Strains and Culture Conditions

The *F. filiformis* heterokaryon strains FL19 (monokaryon L11 and monokaryon L22 were isolated by protoplasmic preparation) and NJ6 (monokaryon 6-3 and monokaryon 6-21 were isolated by protoplasmic preparation) were widely cultivated in the southeast of China. The six strains were obtained from the Fujian Edible Fungi Germplasm Resource Collection Center of China and routinely maintained on potato dextrose agar (PDA) at 25 °C. Mycelia of strains L11, L22, 6-3 and 6-21 were cultured in complete yeast medium (CYM) at 25 °C and was sampled for gene cloning and sequencing. Cultivation of the fruiting body of strain FL19 was performed as per the method described by Tao et al. [17]. The fruiting body was sampled at three major developmental stages: after inoculation, including primordia (PR); young fruiting body (YF); and elongation (EL). The pileus in the elongation stage (ELP) and stipe in the elongation stage (ELS) were also sampled. These fruiting body samples were used for the detection of gene relative expression.

Light and temperature are the most common environmental factors affecting fungi. In order to detect the expression of genes in response to light and temperature, on the mycelia of FL19 the following treatments were performed. For the light treatment, blue light with 3 µmoL·m^−2^·s^−1^ intensity was used to irradiate the mycelia growing on CYM plate for 1 h and 3 h, according to the previous study of Li et al. [18], and the control was that under darkness. For the temperature treatment, four gradients—15 °C (cold stress), 20 °C (low-temperature stress), 25 °C (the optimum temperature, as control), and 30 °C (high-temperature stress)—were used to cultivate *F. filiformis* mycelia after inoculation on CYM plate, according to the study of Lv et al. [19]. Because fungi are often subjected to oxidative stress from growing substrates and mechanical damage from the external environment, the exogenous H_2_O_2_ (5 mM and 10 mM, according to Liu et al. [20]) as the representative of oxidative stress and exogenous MeJA (0.01 mM and 0.02 mM, according to Ren et al. [21]) as the representative of mechanical damage (due to the previous study that showed mechanical damage acts through JA signal [22]) were added into the CYM medium to cultivate *F. filiformis* mycelia. All samples were wrapped in foil, quick-frozen in liquid nitrogen, and stored in a −80 °C freezer for future use.

### 2.2. Bioinformatic Analysis of SPDS and SR in Filamentous Fungi

The sequences of the *Sr* gene (GenBank No.: DAA10591.1) and *Spds* gene (GenBank No.: DAA11489.1) in the model species *S. cerevisiae* were used to identify the corresponding orthologous genes by local BLAST in the *F. filiformis* L11 genome (GenBank Accession No. APIA00000000.1; BioProject: PRJNA191865), 6-3 genome (GenBank Accession No. GCA_011800155.1), and *N. crassa* OR74A genome (GenBank Accession No. GCA_000182925.2; BioProject: PRJNA13841).

The gene structures of *Spds* and *Sr* were visualized using GSDS 2.0 online software (http://gsds.gao-lab.org/, accessed on 5 September 2022), and their conserved domains were predicted by InterProScan online software (http://www.ebi.ac.uk/Tools/pfa/iprscan/, accessed on 7 September 2022). The phylogenetic tree was constructed by MEGAX software with the Neighbor-Joining method and examined by bootstrap testing with 1000 repeats [23].

### 2.3. Cloning and Sequencing of Chimeric FfSpdsSr Transcript in F. filiformis

Total RNA from *F. filiformis* mycelium and fruiting body samples was extracted using E.Z.N.A.^®^ Plant RNA Kit (Omega Bio-Tek, Norcross, GA, USA), and cDNA was reverse transcribed using TransScript^®^ One-Step gDNA Removal and cDNA Synthesis SuperMix Kit (TransGen Biotech, Beijing, China). According to the cDNA sequence of the chimeric *FfSpdsSr* in the *F. filiformis* L11 genome, its full-length ORF was obtained by PCR amplification and sequencing, using the primers designed at both ends of the ORF of *FfSpdsSr* (completely includes the full-length ORF). The two primer pairs FfSpds-qF/R and FfSr-qF/R, which were designed in both SPDS and SR conserved domains, respectively, were used to detect the relative expression level of the chimeric *FfSpdsSr* gene by real-time quantitative PCR (RT-qPCR). All the primers were designed by PrimerQuest Tool online software (https://sg.idtdna.com/PrimerQuest/Home/Index, accessed on 21 September 2022) and are listed in Supplementary Material Table S1. RT-qPCR was performed using a CFX96 Real-Time PCR Detection System (Bio-Rad, Hercules, CA, USA). RT-qPCR amplification was performed using PerfectStart^®^ Uni RT&qPCR Kit (TransGen Biotech, Beijing, China), according to the method of Tao et al. [24]. According to the study of Yang et al. [25], *RNB*, *V*-*ATP*, and *β-TUB* were used as stable internal control genes for the normalization of the RT-qPCR in this study. The relative expression levels of the tested genes were determined according to the 2^−∆∆Ct^ method.

### 2.4. RNA-Seq Data Analyses

The expression correlation of *NcSpds* and *NcSr* in *N. crassa* was analyzed using multiple RNA-Seq data, which was downloaded from the Gene Expression Omnibus (GEO) database (https://www.ncbi.nlm.nih.gov/geo/, accessed on 25 September 2022). The total 15 RNA-Seq datasets were divided into four groups to conduct statistical analyses, according to the sample types: group 1, the different nutritional resources (GSE35227, GSE44100, GSE44673, GSE52316, GSE60986, GSE68517, GSE42692 and GSE51091); group 2, the different development stages (GSE41484); group 3, the different stresses (GSE53013, GSE52153, GSE53534 and GSE61949); and group 4, the different strains (GSE60004 and GSE45406), as well as the whole group, including all of the above samples. The RPKM values of *NcSpds* and *NcSr* were obtained from the above RNA-Seq data in each experimental sample. The correlation of RPKM values of *NcSpds* and *NcSr* genes was analyzed using Graphpad Prism 9 software.

### 2.5. Statistical Analysis

In this study, the results of RT-qPCR experiments were carried out with three independent biological replicates to ensure that the trends and relationships observed were reproducible. The error bars indicate the standard deviation (SD) from the mean of triplicate samples. The significance of the data was analyzed by using one-way ANOVA and multiple comparison tests. 

## 3. Results

### 3.1. Different Gene Structures of Spermidine Synthase and Saccharopine Reductase in F. filiformis and in N. crassa

**Figure 1:**
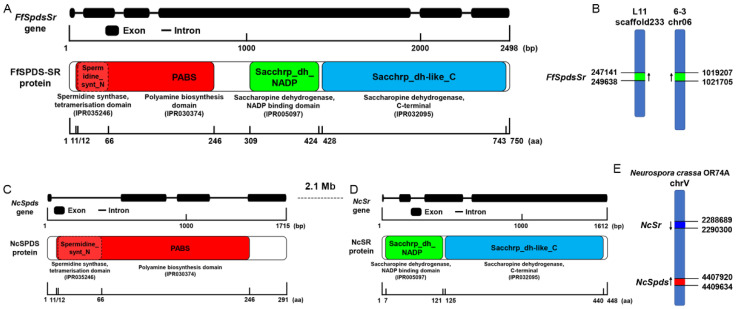
Gene structures and protein conserved domains of *FfSpdsSr* in *Flammulina filiformis* and *NcSpds* and *NcSr* in *Neurospora crassa*. (**A**) Gene structures and protein conserved domains of *FfSpdsSr* in *F. filiformis*. Thick lines represent exons and thin lines represent introns. (**B**) The location of the *FfSpdsSr* gene in the genome of *F. filiformis* strains L11 and 6-3. The green rectangles represent the region of the *FfSpdsSr* gene on the chromosome. The black arrows indicate the ORF direction of the *FfSpdsSr* gene. (**C**) Gene structures and protein conserved domains of *NcSpds* in *N. crassa*. Thick lines represent exons and thin lines represent introns. (**D**) Gene structures and protein conserved domains of *NcSr* in *N. crassa*. Thick lines represent exons and thin lines represent introns. (**E**) The blue rectangle and red rectangle represent the region of *NcSr* and *NcSpds* genes on the chromosome in *N. crassa*, respectively. The black arrows represent the ORF direction of *NcSpds* and *NcSr* genes.

According to the lysine biosynthesis pathway in *S. cerevisiae*, the saccharopine dehydrogenase *LYS9* was used for local BLAST in the genome of *F. filiformis* L11, and its orthologous gene was identified in *F. filiformis* L11 with the ID number of *gene2942* (GenBank No.: OQ378313, Table S2). The length of the *gene2942* is 2498 bp, and it contains six exons and five introns (Figure 1A). Its full-length ORF with 2253 bp encodes a protein with 750 amino acids (aa). By predicting the conserved domain of the protein encoded by *gene2942*, we found that it contains two major conserved domains (Figure 1A): the polyamine biosynthesis domain (IPR030374) in the N-terminal and the saccharopine dehydrogenase domain in the C-terminal. The N-terminal polyamine biosynthesis domain is exactly the spermidine synthase tetramerization domain (IPR035246). The C-terminal saccharopine dehydrogenase domain includes the saccharopine dehydrogenase NADP binding domain (IPR005097) and the saccharopine dehydrogenase C-terminal domain (IPR032095), and it acts as saccharopine reductase in the penultimate step of the lysine biosynthesis pathway named by Liu et al. [26] in *F. filiformis*. It means that *gene2942* is a natural chimeric protein of spermidine synthase (SPDS) and saccharopine reductase (SR), named FfSPDS-SR. Additionally, its fourth exon, as the longest one (1433 bp) in which no termination codon appeared, acts as a bridge between *Spds* and *Sr* to form a natural fusion gene. Theoretically, FfSPDS-SR has the function of both spermidine synthase, which is responsible for catalyzing the biosynthesis of the spermidine, and saccharopine reductase, which is responsible for catalyzing the biosynthesis of the saccharopine as the penultimate intermediate in the biosynthesis of fungal lysine.

To investigate whether there are other separate copies of *Spds* or *Sr*, we performed the local BLAST in the whole genome of *F. filiformis* strain L11 using the conserved domain region of *Spds* or *Sr*, respectively. The results showed that there is the only one copy that is chimeric *FfSpdsSr* (*gene2942*), located at the 247141-249638 bp of the scaffold233 in the genome of *F. filiformis* strain L11 (Figure 1B). The unique copy of chimeric *FfSpdsSr* also exists in the genomes of the other *F. filiformis* monokaryon strains, such as W23, L22, 6-3, and 6-21. In the genome of strain 6-3, obtained by the third-generation sequencing technology Pacbio [27], the unique chimeric *FfSpdsSr* was found to be located at the 1019207-1021705 bp of chromosome 06 (Figure 1B). This suggests that the unique and chimeric *SpdsSr* exists in *F. filiformis* universally.

In the same way, the conserved domain regions of SPDS and SR were used for local BLAST in the genome of *N. crassa* OR74A, respectively. Two separate orthologous proteins NCU06727 and NCU03748 were obtained. The length of the NCU06727 gene is 1715 bp and contains four exons and three introns (Figure 1C). Its full-length ORF with 876 bp encodes a protein with 291 aa, which contains only one main conserved domain: the polyamine biosynthesis domain (IPR030374). The exact domain of NCU06727 is the spermidine synthase tetramerization domain (IPR035246); thus, we renamed NCU06727 as NcSPDS (Figure 1C). The length of the NCU03748 gene is 1612 bp and contains four exons and three introns (Figure 1D). Its full-length ORF with 1347 bp encodes a protein with 448 aa, which contains only the saccharopine dehydrogenase domains. The detailed saccharopine dehydrogenase domains in NCU03748 include the saccharopine dehydrogenase NADP binding domain (IPR005097) and the saccharopine dehydrogenase C-terminal domain (IPR032095) (Figure 1D). Because that saccharopine dehydrogenase is in charge of the penultimate step of the fungal lysine biosynthesis and also called saccharopine reductase (SR) previously, we renamed NCU03748 as NcSR. 

The polyamine biosynthesis domain (IPR030374) and the spermidine synthase tetramerization domain (IPR035246) are located at 11-246 aa and 12-66 aa in FfSPDS-SR and NcSPDS, respectively. Additionally, the saccharopine dehydrogenase NADP binding domain (IPR005097) and the saccharopine dehydrogenase C-terminal domain (IPR032095) are separated by 3 aa in both FfSPDS-SR and NcSR. The chimeric FfSPDS-SR protein is 11 more aa than NcSPDS plus NcSR, so these 11 aa region links SPDS and SR in chimeric FfSPDS-SR protein.

According to the genome of *N. crassa* OR74A, the *NcSr* gene was located at the 2288689-2290300 bp of the chromosome chrV (CM002240.1) (Figure 1E), while the *NcSpds* gene was located at the 4407920-4409634 bp of the chromosome chrV (CM002240.1) (Figure 1E). The *NcSpds* gene and *NcSr* gene are located on the same chromosome (chrV), but they are 2.1 Mb apart and have the opposite direction (Figure 1C,D). *NcSpds* and *NcSr* are two physically separate, relatively independent genes, with no forming of a chimeric gene.

### 3.2. The Chimeric FfSpdsSr Gene Presents in Heterokaryon of F. filiformis with Conserved and Unitary Transcript

**Figure 2:**
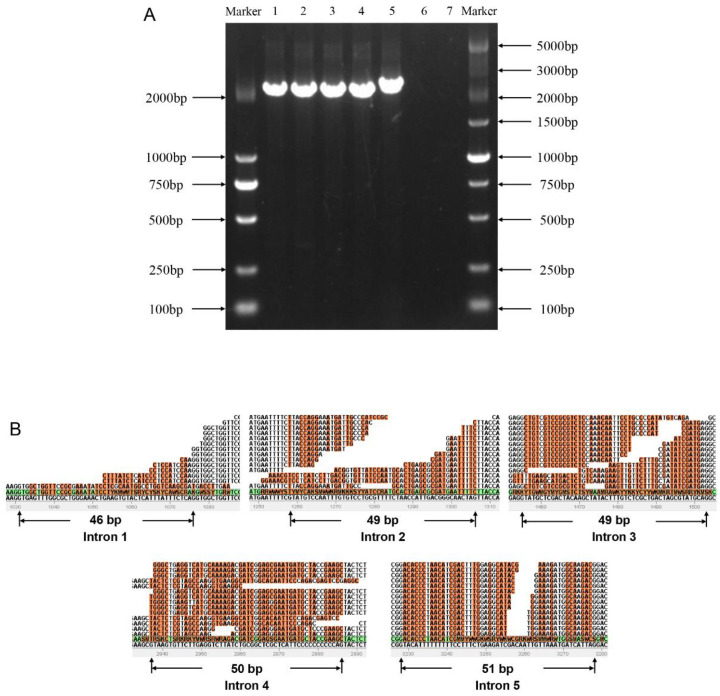
Validation of the full-length ORF and unitary transcript of *FfSpdsSr* gene in *F. filiformis*. (**A**) Full-length ORF amplification results of *FfSpdsSr* gene in *F. filiformis*. Numbers 1–4 represent the full-length ORF of chimeric *FfSpdsSr* amplified from cDNA of monokaryon L11 (isolated from dikaryon FL19), L22 (isolated from dikaryon FL19), 6-3 (isolated from dikaryon NJ6), and 6-21 (isolated from dikaryon NJ6) in *F filiformis*, respectively. Number 5 represents the full length of gDNA of *FfSpdsSr* in *F. filiformis* strain L11. Numbers 6–7 represent the PCR results using cDNA as template and the combined primers (the beginning of ORF of *NcSpds* as forward primer and the end of ORF of *NcSr* as reverse primer) in *N. crassa* strain, FGSC#4200 and FGSC#2489. The markers 2000 and 5000 are shown on the side of the electropherogram. (**B**) Alternative splicing analysis of *FfSpdsSr* in *F. filiformis*. The orange parts represent the intron region of *FfSpdsSr*.

In order to determine whether the alleles of the *FfSpdsSr* gene exist in different forms in two different nuclei of *F. filiformis* heterokaryon strain, we used two *F. filiformis* strains FL19 and NJ6, which are widely cultivated in the southeast of China. The heterokaryon FL19 separated two monokaryon L11 and monokaryon L22, while the heterokaryon NJ6 separated two monokaryon 6-3 and monokaryon 6-21. The genome sequences of the *FfSpdsSr* gene in L11, L22, 6-3, and 6-21 were performed multiple sequence alignment, the results showed that the length of the alleles of the *FfSpdsSr* gene in four strains is 2494-2498 bp, and the similarity was 99.3% among them (SNP site variation). Further, the full-length ORF of the *FfSpdsSr* gene was also performed PCR amplification and sequencing, using the primers designed at both ends of the ORF of *FfSpdsSr* (completely includes the ORF). The unique transcript (chimeric ORF of the chimeric *FfSpdsSr* gene) was confirmed by the single PCR band and the Sanger sequencing results in each of L11, L22, 6-3, and 6-21 strains (Figure 2A). Meanwhile, a single PCR band was also amplified for the full-length ORF of *FfV-ATP* and *Ffβ-TUB* in L11 and L22 as the positive control (Figure S1). The unique transcript sequence of *FfSpdsSr* is consistent with the results predicted by the genome, meaning that the exons, introns, and their sites of the *FfSpdsSr* gene are accurate. In addition, in order to explore whether the *FfSpdsSr* gene forms different alternative splicing variants during transcription through alternative splicing, we mapped the raw reads of the transcriptome from the different developmental stages on the *FfSpdsSr* gDNA sequence using ZOOM software. The mapping results showed that there are a total of five introns in the *FfSpdsSr* gene, which all showed a 5′ (GT) and 3′ (AG) splice site in four introns. Importantly, none of the reads were detected to be derived from adjacent exons and introns, meaning that all introns of *FfSpdsSr* were clipped out during transcription (Figure 2B). Together with the results of full-length ORF cloning and sequencing, this suggests that the gene *FfSpdsSr* has only one transcript: the fused CDS of *SpdsSr*.

Similarly, the beginning of the ORF of *NcSpds* as the forward primer and the end of the ORF of *NcSr* as the reverse primer were designed to test whether the chimeric *SpdsSr* transcript exists in *N. crassa*. The PCR, using cDNA as the template, was performed in the two strains (monokaryon FGSC#2489 and monokaryon FGSC#4200, which can mate into a heterokaryon strain) of *N. crassa.* There was no PCR band using the above primer pair in two *N. crassa* strains; however, a single PCR band was amplified for the full-length ORF of *NcV-ATP* and *Ncβ-TUB* as the positive control (Figure S1), confirming that *NcSpds* and *NcSr* do not form chimeric transcripts at the RNA level.

### 3.3. Gene Structure and Phylogeny Evolution of SPDS and SR in Fungi

**Figure 3:**
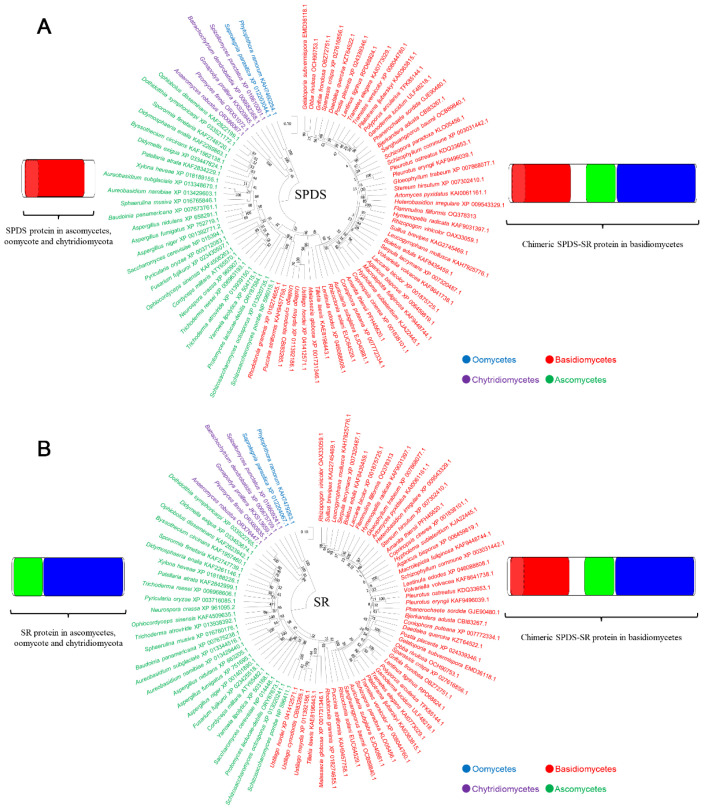
Phylogenetic analysis of SPDS and SR in fungi. (**A**) Phylogenetic trees based on SPDS domain in fungi, including basidiomycetes, ascomycetes, chytrid fungi, and oomycetes. (**B**) Phylogenetic trees based on SR domain in fungi, including basidiomycetes, ascomycetes, chytridiomycetes, and oomycetes.

In order to analyze whether chimeric *SpdsSr* is also present in fungi other than *F. filiformis*, we investigated 48 basidiomycetes, 27 ascomycetes, five chytridiomycetes, and two oomycetes; their complete genome sequences can be obtained in NCBI or JGI database. The conserved domain regions of SPDS and SR were used for local BLAST to obtain the orthologous gene of chimeric *SpdsSr* or the two separate *Spds* and *Sr* genes in the above species. The results showed that chimeric *SpdsSr* was found in all 48 basidiomycetes, while the separate *Spds* and *Sr* genes were found in all 27 ascomycetes, five chytridiomycetes, and two oomycetes. Furthermore, the phylogenetic trees were constructed based on the conserved SPDS and SR domain regions. Both the phylogenetic tree based on the SPDS domain (Figure 3A) and the phylogenetic tree based on the SR domain (Figure 3B) showed that 82 species are clustered into four groups: basidiomycetes, ascomycetes, chytridiomycetes, and oomycetes, which correspond to four phyla of fungi. This also suggests that the formation of chimeric *SpdsSr* is closely related to the evolution of fungi.

### 3.4. Expression Patterns of the Chimeric Gene SpdsSr in F. filiformis

**Figure 4:**
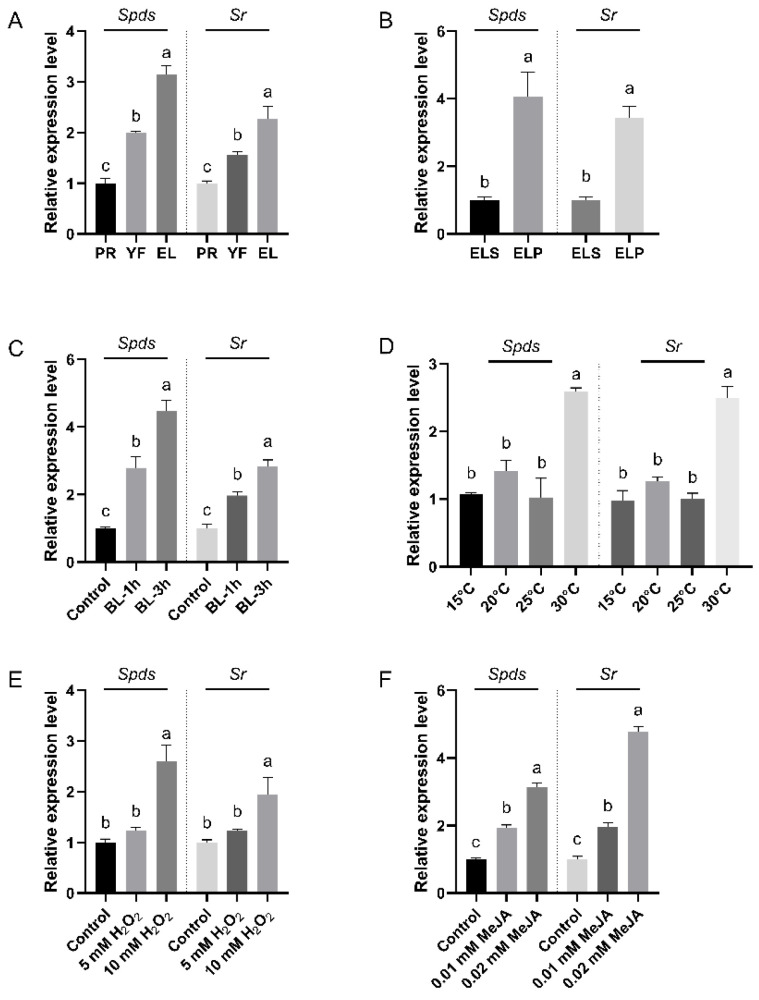
Relative expression levels of *FfSpdsSr* gene in different developmental stages, different tissues, and under different environmental factors in *F. filiformis*. (**A**) Relative expression levels of *FfSpdsSr* in different developmental stages of strain FL19. PR: primordia; YF: young fruiting body EL: elongation stage. (**B**) Relative expression levels of *FfSpdsSr* in different tissues of strain FL19. ELS: stipe in elongation stage; ELP: pileus in elongation stage. (**C**) Relative expression levels of *FfSpdsSr* under blue light conditions. BL-1h: 1 h under blue light irradiation, BL-3h: 3 h under blue light irradiation. (**D**) Relative expression levels of the *FfSpdsSr* gene of *F. filiformis* in strain FL19 under different temperature gradients, including 15 °C (cold stress), 20 °C (low-temperature stress), 25 °C (the optimum temperature, as control), and 30 °C (high-temperature stress). (**E**) Relative expression levels of *FfSpdsSr* gene of *F. filiformis* in strain FL19 under H_2_O_2_ condition. (**F**) Relative expression levels of *FfSpdsSr* gene of *F. filiformis* in strain FL19 under MeJA condition. (**A**–**F**) Spds represents the RT-qPCR results using the primers FfSpdsF/R designed in the SPDS domain, and Sr represents the RT-qPCR results using the primer FfSrF/R designed in the SR domain. All values are the means ± SD of three independent experiments, and the lowercase letters indicate significant differences among different samples (*p* < 0.05).

In order to explore the roles of the chimeric *FfSpdsSr* gene in the growth and development of *F. filiformis* as well as in response to various environmental stresses, the transcription levels of *FfSpdsSr* were detected in different developmental stages, tissues, common environment factors (light and temperature), and stresses (H_2_O_2_ represents oxidative stress and exogenous MeJA represents mechanical damage signal). To verify whether the expression trend of 5′-end *FfSpds-*region and 3′-end *FfSr*-region of the chimeric gene *FfSpdsSr* was consistent, we designed the RT-qPCR primers FfSpds-qF/R and FfSr-qF/R on the *FfSpds* and *FfSr* conserved domain sequences, respectively. First of all, the expression trends of the *FfSpds*-region and the *FfSr*-region were highly consistent in all of the test samples because they belong to the unitary transcript of *FfSpdsSr*. During the three major developmental stages (PR, YF, EL), the transcription levels of *FfSpdsSr* showed an up-regulated trend from primordia to the elongation stage as the fruiting body grew (Figure 4A). For the stipe and pileus in the elongation stage, the transcription level of *FfSpdsSr* in the pileus (ELP) was about 4-fold higher than that in the stipe (ELS) (Figure 4B). Just as the blue light was usually used to shine *F. filiformis* in the factory, the two time points (1 h and 3 h, according to the previous study by Li et al. [18]) after blue light irradiation were used for the sample and detection. *FfSpdsSr* also up-regulated at 1 h and 3 h after blue light irradiation, and its relative expression level at BL-3h was higher than that at BL-1h (Figure 4C). According to the study of Lv et al. [19], four temperature gradients (15 °C, 20 °C, 25 °C, 30 °C) were used to detect the response expression of *FfSpdsSr*. *FfSpdsSr* showed no significant difference in expression under the 15 °C or 20 °C cultivations (low temperature) compared with the 25 °C cultivation (the optimum temperature), while it showed a 2.5-fold up-regulation under the 30 °C cultivation (high temperature) (Figure 4D). Besides the light and temperature, many cultivation substrates often bring oxidative stress and mechanical damage often occurs. During the growth of *F. filiformis*, we adopted the exogenous addition of H_2_O_2_ (5 mM and 10 mM, according to Liu et al. [20]) and MeJA (0.01 mM and 0.02 mM, according to Ren et al. [21]) as two cases of environmental stress to detect the response expression of *FfSpdsSr*. The RT-qPCR results showed that *FfSpdsSr* significantly up-regulated under 10 mM H_2_O_2_ treatment (Figure 4E) or under 0.01-0.02 mM MeJA treatment (Figure 4F).

### 3.5. Similar Expression Patterns of NcSpds and NcSr in N. crassa

Although *Spds* and *Sr* are two separate and independent genes in ascomycetes, we wondered if there was some correlation between expression and function. Since *N. crassa* is a model ascomycetes species, there have been numerous genome-wide expression profile data under a variety of different conditions; we downloaded, in total, 15 sets of RNA-Seq data from the GEO database, in order to analyze the expression correlation of *NcSpds* and *NcSr*. These 15 sets of RNA-Seq data were gathered from different experimenters around the world and included a total of 218 samples. Although different sequencing depths among RNA-Seq datasets led to a large range of changes in the absolute expression value for each gene, RPKM values of *NcSpds* and *NcSr* were calculated under the same internal standard and can be compared in each sample. Thus, we performed the correlation analysis based on the RPKM values of *NcSpds* and *NcSr* obtained from the above RNA-Seq datasets. 

**Figure 5:**
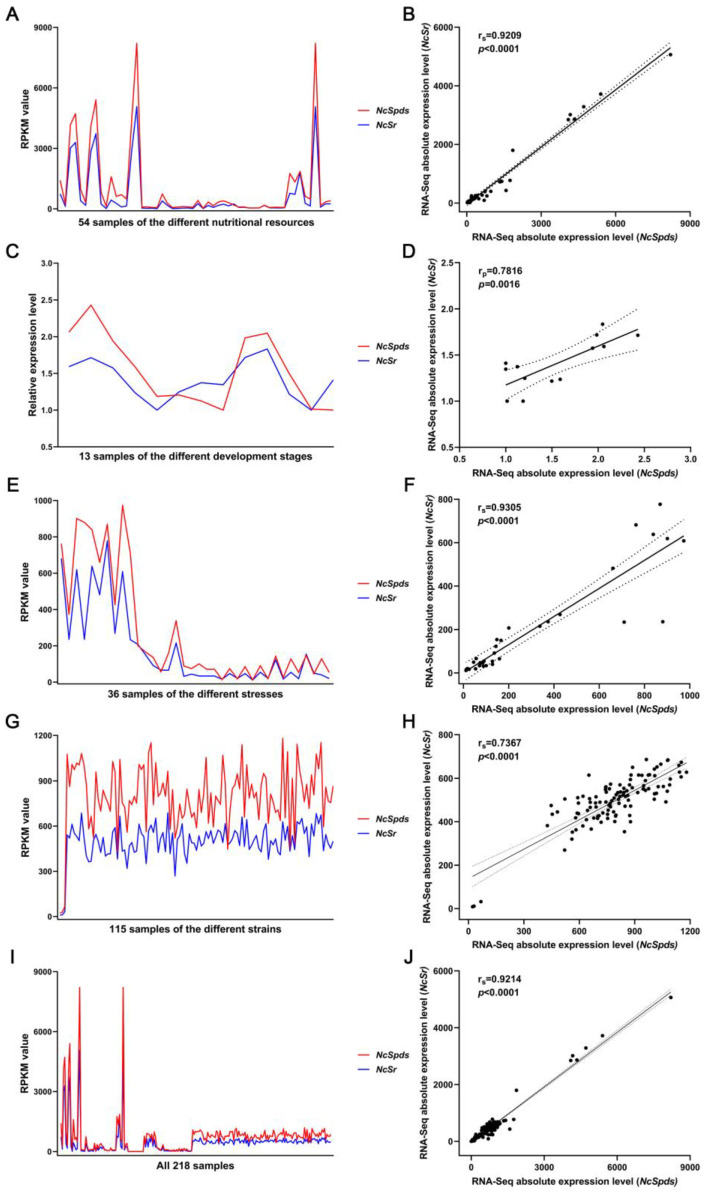
Correlation analyses of expression levels of *NcSpds* and *NcSr* in *N. crassa*. (**A**,**B**) Correlation analyses of expression levels of *NcSpds* and *NcSr* in the different nutritional resources, including avicel, sucrose, no carbon, xylan, barleystraw, cornstraw, rice straw, soybean straw, wheatstraw, pectin, orange peel powder (OPP), L-arabinose, D-glucose, and D-xylose (RNA-Seq data: GSE35227, GSE44100, GSE44673, GSE52316, GSE60986, GSE68517, GSE42692 and GSE51091). (**C**,**D**) Correlation analyses of expression levels of *NcSpds* and *NcSr* in the different development stages, including perithecium wall (PW), centrum parenchyma (CP), asci (AS), paraphyses (PP), and ostiole (OS) (RNA-Seq data GSE41484). (**E**,**F**) Correlation analyses of expression levels of *NcSpds* and *NcSr* under the different stresses, including dimethylsulfoxide (DMSO), staurosporine (STS), dark, light for 15 min, light for 60 min, light for 120 min, light for 240 min, dithiothreitol (DTT, 5 mM), DTT (0.1 mM), H_2_O, tunicamycin (TM, 40 μg/mL), and TM (5 μg/mL) (RNA-Seq data: GSE53013, GSE52153, GSE53534 and GSE61949). (**G**,**H**) Correlation analyses of expression levels of *NcSpds* and *NcSr* in the 115 different strains, including Δ3*β*G, CPL4, and CPL7 and the 112 strains collected in Louisiana [28] (RNA-Seq data: GSE60004 and GSE45406). (**I**,**J**) Correlation analyses of expression levels of *NcSpds* and *NcSr* in all 218 samples from RNA-Seq data. (**A**,**C**,**E**,**G**,**I**) The red lines and the blue lines represent the expression levels of *NcSpds* and *NcSr*, respectively. (**B**,**D**,**F**,**H**,**J**) Correlation analyses were performed on the expression levels of *NcSpds* and *NcSr*. The “r_p_” was the Pearson correlation coefficient and the “r_s_” was the Spearman correlation coefficient. The black dots represent the expression level of *NcSpds* and *NcSr* in each sample. The black dotted line represents the 95% confidence bands of the best-fit line.

In group 1 (Figure 5A), there were a total of 54 samples involved in different nutritional resources, including avicel, sucrose, no carbon, xylan, and straw. In general, RPKM values of *NcSpds* and *NcSr* showed similar patterns under these nutritional conditions, and the correlation coefficient r_s_ value was 0.9209 (*p* < 0.0001) (Figure 5B), which was larger than the threshold of the strong correlation coefficient, 0.8. In Figure 5B, most of the sample points clustered within the 95% confidence bands of the best-fit line. In addition, the RPKM peak of *NcSpds* and *NcSr* mainly appeared in the sample under the sucrose nutrient condition. 

In group 2 (Figure 5C), there were a total of 13 samples involved in the different development stages, including perithecium wall, centrum parenchyma, asci, paraphyses, ostiole, etc. The RPKM values of *NcSpds* and *NcSr* showed slightly similar patterns under these development stages, and the correlation coefficient r_p_ value was 0.7816 (*p* = 0.0016) (Figure 5D), which was slightly lower than the threshold of strong correlation coefficient, 0.8, but still higher than the threshold of acceptable correlation coefficient, 0.6. In addition, the RPKM peak of *NcSpds* and *NcSr* mainly appeared in the sample around sexual development.

In group 3 (Figure 5E), there were a total of 36 samples involved in the different stresses, including dimethylsulfoxide, staurosporine, light, dithiothreitol, tunicamycin, etc. In general, the RPKM values of *NcSpds* and *NcSr* showed highly similar patterns under these stresses, and the correlation coefficient r_s_ value was 0.9305 (*p* < 0.0001) (Figure 5F), which was higher than the threshold of strong correlation coefficient 0.8. In Figure 5F, most sample points are clustered within the 95% confidence bands of the best-fit line. The RPKM peak of *NcSpds* and *NcSr* mainly appeared in the sample under dimethylsulfoxide stress.

In group 4 (Figure 5G), there were a total of 115 different mutant strain samples. The RPKM values of *NcSpds* and *NcSr* showed a slightly similar pattern under these strains, and the correlation coefficient r_s_ value was 0.7367 (*p* < 0.0001) (Figure 5H), less than 0.8 but still higher than the threshold of acceptable correlation coefficient, 0.6. When we put all 218 samples together for analysis (Figure 5I), the absolute expression of *NcSpds* and *NcSr* showed a very similar trend, and the correlation coefficient r_s_ value was 0.9214 (*p* < 0.0001) (Figure 5J), suggesting that *NcSpds* and *NcSr* were strongly correlated at the transcription level. 

## 4. Discussion

Gene fusion means that two or more independent genes are placed in one set of ORFs via chromosomal rearrangement and combination, which is under the same regulatory sequence, including promoter and terminator. The result of gene fusion is to form a chimeric gene, and the expression product of the chimeric gene is chimeric protein. In most cases, gene fusion can result in abnormal sequences, disordered expression of certain genes, and protein function deactivation, which can lead to or promote the development of tumors. In past studies, a natural chimeric gene was mostly found in cancer cells, so many chimeric genes have been established as important biomarkers and therapeutic targets in multiple cancer types [29]. To date, it has been rarely reported in normal plant, animal, and fungal cells. Thus, in this study, the discovery of the natural chimeric gene SPDS-SR in basidiomycetes such as *F. filiformis*, as well as the confirmation of its single copy and unique variant spliceosome, are of great biological significance for the study of gene evolution. This is because gene fusion events can serve as phylogenetic markers [30].

In the 27 ascomycetes investigated in this paper, SPDS and SR did not fuse but existed independently as two genes. The cis-regulatory sequences of the promoters of these two genes are also different. However, from 15 sets of expression profile data, the expression patterns of *Spds* and *Sr* showed a highly positive correlation. Due to these expression profile data being derived from high-throughput RNA-Seq under the different sample types, different treatments, different strains, and different experiment times, the positive correlation obtained by statistics is highly reliable. It also suggests that the transcription of *Spds* and *Sr* (although they are not fused) tended to synchronize in ascomycetes, especially in some special development periods and under some environmental factor treatments.

The similar expression patterns of *Spds* and *Sr* in ascomycetes may be due to the fact that they are regulated by the same transcription factor (although there are no reports about this). The fusion of the two genes may improve the regulation efficiency [6]. Another possibility is that the fungi require the combined action of polyamines and lysine during the special growth and development stages or in response to some environmental changes, as this study found that high expression of *NcSpds* and *NcSr* appeared during sexual development or under osmotic stress (DMSO treatment), etc. Coincidentally, *FfSpdsSr* also significantly up-regulated during the pileus’ rapid development or under several environmental stressors, including blue light, high temperature, H_2_O_2_, and MeJA treatment. Therefore, co-expression of the two genes encoding synthetase is required in ascomycetes. While in basidiomycetes, these two genes directly form the one chimeric gene. The occurrence of *SpdsSr* forming a chimeric gene also suggests that spermidine and lysine biosynthesis are linked directly. Previous studies have proved the involvement of spermidine as an antioxidant against the potential abiotic-stress-derived oxidative damage [31]. Spermidine, as a type of signaling molecule, also play important roles in growth and development and the defense against biotic and abiotic stresses, while lysine is directly involved in many biological processes, such as constituent protein structure, stress response, secondary metabolites biosynthesis, etc. [32]. Both spermidine and lysine can promote growth, cell division, DNA replication, and cell differentiation through interacting with nucleic acids, proteins, and phospholipids to regulate the physical and chemical properties of the membrane, the structure and function of nucleic acids, and the activity of enzymes. It has been reported that the deficiency of polyamines and lysine can cause an imbalance of ROS homeostasis and a disorder of physiological function. 

Gene fusion of related functions can improve the efficiency of biochemical reactions and signal transduction, as well as promote the coordinated regulation of their expression [33]. Based on the analysis of the cis-elements of the promoter of the *SpdsSr* gene in basidiomycetes and *Spds* and *Sr* genes in ascomycetes and the summary of the existing expression profile data, it can be seen that *SpdsSr*, *Spds*, and *Sr* are mainly found in the rapid development period of fruiting bodies (such as the rapid elongation stage of *F. filiformis*); in the tissue parts of sexual reproductive organs (such as the pileus development and the basidiospore formation); and when multiple environmental factors, including blue light, high-temperature stress, oxidative stress (such as H_2_O_2_ treatment), and hormonal changes (such as MeJA treatment), are up-regulated. This suggests that *SpdsSr* (or the combination of spermidine and lysine) may be mainly involved in the growth and development process of fungi and play important roles in coping with environmental stress. This is also supported by the fact that the fusion of SPE3-LYS9 in *C. neoformans* is important for virulence and survival in vivo, and that the spe3-lys9 mutants were avirulent and unable to survive in vivo [7].

From an evolutionary point of view, ascomycetes seem to be more junior and basidiomycetes more advanced. Basidiomycetes (especially large edible fungi) have the characteristics of fruiting bodies, such as a stipe and pileus. Meanwhile, during the development of fruiting bodies, basidiomycetes are more susceptible to changes in environmental factors such as temperature and light. These environmental factors also regulate the changes in the shapes and colors of basidiomycetes from time to time, so that fungi can better adapt to the living environment. The functional convergent evolution of the fused *SpdsSr* suggests the evolutionary advantages of basidiomycetes in utilizing spermidine and lysine. At present, there is no sufficient evidence as to whether *SpdsSr* is to meet the growth and development and cope with the changes of environmental factors. However, the phenomenon of the natural fusion form and co-expression pattern in basidiomycetes should indeed attract the interest of fungal researchers. It is also worth attention to further reveal the biological significance of this fusion phenomenon from heredity and evolution.

## 5. Conclusions

The spermidine synthetase and saccharopine reductase encoding genes form a natural chimeric gene, *FfSpdsSr,* in *F. filiformis*, which is also found to present in the other 48 tested basidiomycetes, while the two separate *Spds* and *Sr* genes independently present in *N. crassa* and are also found to present in the other tested 27 ascomycetes, five chytridiomycetes, and two oomycetes. In *F. filiformis*, the full-length ORF PCR results and no alternative splicing variant of *FfSpdsSr* confirms that the unique chimeric *FfSpdsSr* transcript functions in *F. filiformis*. *FfSpdsSr* mainly up-regulated in the elongation stage and pileus development of *F. filiformis*, as well as under the blue light, high-temperature, H_2_O_2_, and MeJA treatments. Interestingly, the separate *NcSpds* and *NcSr* in *N. crassa* were found to share highly similar co-expression patterns in the total 15 RNA-Seq datasets, including 218 samples with different strains and different nutritional and environmental condition treatments. This implies that the fusion of *Spds* and *Sr* may have great biological significance and meet evolutionary needs.

## Data Availability

All experimental data in this study will be made available upon reasonable request from readers. Publicly available datasets were analyzed in this study. Data is available at NCBI GEO: GSE35227, GSE44100, GSE44673, GSE52316, GSE60986, GSE68517, GSE42692, GSE51091, GSE41484, GSE53013, GSE52153, GSE53534, GSE61949, GSE60004 and GSE45406. This data can be found here: https://www.ncbi.nlm.nih.gov/geo/ (accessed on 25 September 2022).

## Supplementary Materials

The following supporting information can be downloaded at: https://www.mdpi.com/article/10.3390/jof9030352/s1, Figure S1: PCR of the full-length ORF of internal control genes in *F. filiformis* and *N. crassa*; Table S1: The primers used in this study; Table S2: The gene accession number used in this study.

## Abbreviations

SPDS	Spermidine synthetase
SR	Saccharopine reductase
AAA	α-Aminoadipate
CYM	Complete yeast medium
PDA	Potato dextrose agar
PR	Primordia
YF	Young fruiting body
EL	Elongation stage
ELP	Pileus in elongation stage
ELS	Stipe in elongation stage
JA	Jasmonic acid
MeJA	Methyl jasmonate
RT-qPCR	Real-Time quantitative PCR
SD	Standard deviation
BL-1h	1 h under blue light irradiation
BL-3h	3 h under blue light irradiation
GEO	Gene Expression Omnibus
