# Supplementary material for: Spermidine Synthase and Saccharopine Reductase Have Co-Expression Patterns Both in Basidiomycetes with Fusion Form and Ascomycetes with Separate Form
Source: J Fungi (Basel). 2023 Mar 14;9(3):352. doi: 10.3390/jof9030352 (PMC10051792; doi:10.3390/jof9030352)
Supplement: Supplementary file 1 [file jof-09-00352-s001.zip › jof-2167827-supplementary.pdf]

Supplementary materials:

Spermidine Synthase and Saccharopine Reductase have Co-expression Patterns Both in Basidiomycetes with Fusion Form and Ascomycetes with Separate Form

Yayong Yang [1,2], Lei Shi [1,2], Xinyu Xu [1,2], Jin Wen [1,2], Tianyue Xie [1,2], Hui Li [3], Xiaoyu Li [1,2], Mengyu Chen [1,2], Xinyi Dou [1,2], Chengjin Yuan [1,2], Hanbing Song [1,2], Baogui Xie [2], Yongxin Tao [1,2,*]

[1] College of Horticulture, Fujian Agriculture and Forestry University, Fuzhou 350002, China
[2] Mycological Research Center, Fujian Agriculture and Forestry University, Fuzhou 350002, China
[3] Institute of Cash Crops, Hebei Academy of Agriculture and Forestry Sciences, Shijiazhuang 050051, China
* Correspondence: taoyongxinmuse@163.com; Tel.: +86-0591-83789281 (Y.T.)

Table S1. The primers used in this study.

Gene symbol	Primer sequence (5′ to 3′)	Description
FfSpdsSr-F<br>FfSpdsSr-R	ATGGCACCTTTATCTCATCCC<br>CACTCAGAGAACTCGCTCAAC	*FfSpdsSr* full-length ORF cloning
NcSpds-F<br>NcSr-R	GATGTCCGAAATTGCTCACC<br>TCGCATTGAGCTTAGGAGATG	Verifying whether *NcSpds* and *NcSr* transcript was fused
FfSpds-qF<br>FfSpds-qR	AAGCCATACTTCCAGCTTCTTC<br>GGCGTATTCAGAGACAGGAAAG	Detecting expression level of *FfSpds* conserved domain by RT-qPCR
FfSr-qF<br>FfSr-qR	GGACACCCTAACATCGACTTTG<br>GTCACAGATCTCCTTGCTGTATG	Detecting expression level of *FfSr* conserved domain by RT-qPCR
β-TUB-qF<br>*β*-TUB-qR	CAAATGCAGAACGTCCAGAAC<br>GTGAACTCCATCTCGTCCATAC	Internal control gene for RT-qPCR
RNB-qF<br>RNB-qR	TTGTTCCTCTGTGTTCTCATCTC<br>GACATCCTTCGTGGACCAATAG	Internal control gene for RT-qPCR
V-ATP-qF<br>V-ATP-qR	GCAGAATGGTATGAGCGAGTAT<br>TCTGGGACAGCTTGAACATAAG	Internal control gene for RT-qPCR
Ff*β*-TUB-F<br>Ff*β*-TUB-R	GAGCGCGATTTGACTTTCTTC<br>ACAGAGTCTACTCCTCGTCAG	Housekeeping gene for PCR
FfV-ATP-F<br>FfV-ATP-R	ACATCTGCATATACCACGACAA<br>GAGCATTACACCTACTCAACCA	Housekeeping gene for PCR
Nc*β*-TUB-F<br>Nc*β*-TUB-R	TCATCACCAAACCGTCAAGAT<br>GATTTACTCCTCGCCCTCAAG	Housekeeping gene for PCR
NcV-ATP-F<br>NcV-ATP-R	CGACCTTCTCTGAAACCTACCT<br>GCGCTCGTTCACTCATCAAT	Housekeeping gene for PCR

Table S2. The gene accession number used in this study.

Gene symbol	Gene name	Accession number in NCBI
FfSpdsSr	Spermidine synthase and saccharopine reductase	OQ378313
β-TUB	Tubulin beta	OP354402
RNB	Exosome complex exonucleaseDIS3/RRP44	OP354411
V-ATP	V-type H^+-transporting ATPase subunit A	OP354412
NcSpds	Spermidine synthase	NCU06727
NcSr	Saccharopine dehydrogenase	NCU03748

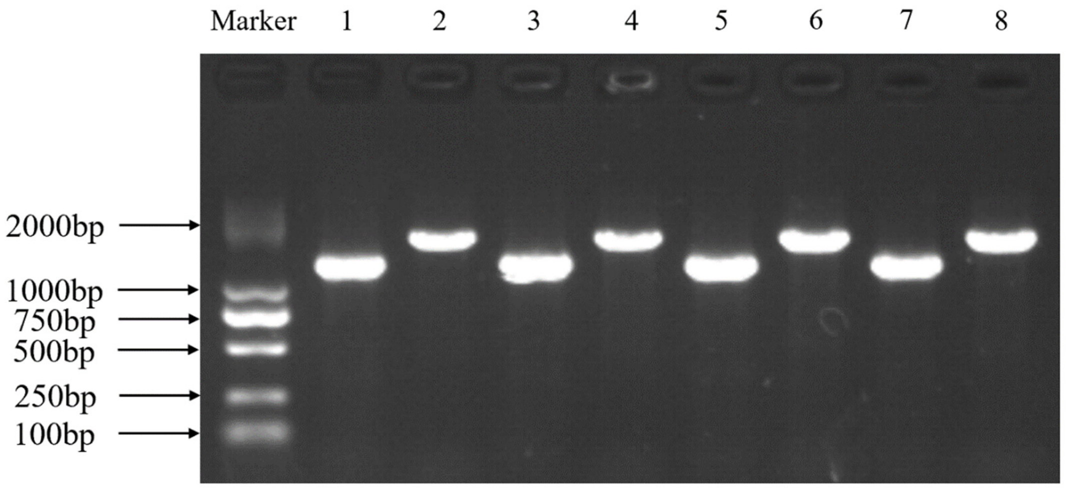

Figure S1. PCR of the full-length ORF of internal control genes in *F. filiformis* and *N. crassa*. Numbers 1 and 3 represent the full length ORF of *β-TUB* amplified from cDNA of L11 and L22 in *F. filiformis*. Numbers 5 and 7 represent the full length ORF of *β-TUB* amplified from cDNA of FGSC#4200 and FGSC#2489 in *N. crassa*. Numbers 2 and 4 represent the full length ORF of *V-ATP* amplified from cDNA of L11 and L22 in *F. filiformis*. Numbers 6 and 8 represent the full length ORF of *V-ATP* amplified from cDNA of FGSC#4200 and FGSC#2489 in *N. crassa*.
